# Patient reported respiratory symptoms and lung radiation doses 11 years after loco-regional breast cancer radiation therapy in the DBCG RT Nation Study

**DOI:** 10.2340/1651-226X.2025.43973

**Published:** 2025-10-01

**Authors:** Maja Bendtsen Sharma, Lasse Hindhede Refsgaard, Elisabeth Bendstrup, Emma Skarsø Buhl, Robert Zachariae, Rasmus Blechingberg Friis, Ingeborg Farver-Vestergaard, Stine Sofia Korreman, Birgitte Vrou Offersen

**Affiliations:** a Department of Oncology, Aarhus University Hospital, Aarhus, Denmark; b Danish Centre for Particle Therapy, Aarhus University Hospital, Aarhus, Denmark; c Department of Clinical Medicine, Aarhus University, Aarhus, Denmark; d Centre for Rare Lung Diseases, Department of Respiratory Diseases and Allergy, Aarhus University Hospital, Aarhus, Denmark; e Department of Psychology and Behavioural Sciences, Aarhus University, Aarhus, Denmark; f Department of Medicine, Lillebaelt Hospital, Vejle, Denmark; g Department of Regional Health Research, University of Southern Denmark, Odense, Denmark; h Department of Experimental Clinical Oncology, Aarhus University Hospital, Aarhus, Denmark

**Keywords:** Adjuvant loco-regional radiation therapy, breast cancer, late effects, pulmonary late effects

## Abstract

**Background and purpose:**

The Danish Patient Association of Late Effects has received numerous inquiries from breast cancer (BC) survivors suspecting that adjuvant radiotherapy (RT) was the reason for respiratory symptoms. This study investigated patient-reported respiratory symptoms after locoregional RT and their association with ipsilateral lung radiation dose.

**Patient/material and methods:**

Patient-reported outcomes (PROs) and RT plans from BC patients treated at a single institution over 2008–2016 were collected. PROs included dyspnoea (EORTC QLQ-C30), cough (PRO-CTCAE), smoking and comorbidities. RT dose-volume metrics were registered including ipsilateral mean lung dose (MLD), and volumes receiving 5 Gy (V5) and 20 Gy (V20). Patients were stratified into MLD tertiles (‘low’, ‘intermediate’, ‘high’) and compared. Additionally, responders were dichotomised by dyspnoea and cough scores (‘low’ vs ‘high’), and dose metrics were compared between symptom groups.

**Results:**

Of 1,011 questionnaire distributed, 490 (49%) were completed and analysed. Median age was 65.8 years (interquartile range [IQR] 58.8;73.4), median time from RT to questionnaire was 11.1 years (IQR 8.9;13.2). Overall MLD was 12.9Gy (standard deviation [SD] 2.8). Any degree of dyspnoea was reported by 203 (41%) and any degree cough was reported by 175 (37%). No differences in dyspnoea/cough scores between MLD groups were found. MLD was 13.0Gy (SD 2.7) in the low dyspnoea group and 12.0Gy (SD 3.0) in the high dyspnoea group, (*p* = 0.04). MLD was 13.0 Gy (SD 2.7) in the low cough group and 12.5Gy (SD 3.1) in the high cough group (*p* = 0.23). The same pattern was found for V5lung and V20lung.

**Interpretation:**

No associations between lung dose and patient-reported respiratory symptoms were found for node-positive BC patients 11 years after RT.

## Introduction

Breast cancer (BC) is the most frequent non-skin cancer in Danish women with an incidence of approximately 5,200 cases annually [1]. With mammography screening, improved imaging and pathology evaluation in addition to intensified multi-modal treatment, survival after BC has increased considerably over the recent decades, resulting in a growing population of long-term survivors at risk of late effects [2–5]. In Denmark, adjuvant BC radiotherapy (RT) is administered to approximately 3,500 patients annually, 35% of whom receive loco-regional RT for high-risk BC.

Patient-reported outcome measures (PROMs) assess perceived effects of disease, systemic therapy or RT on tissue function [6]. The European Organisation for Research and Treatment of Cancer (EORTC) has developed validated, multilingual questionnaires to evaluate general and organ-specific health-related quality of life (HRQoL). The EORTC QLQ-C30 is the most widely used tool for assessing general HRQoL, including pulmonary, gastrointestinal and cognitive functions. While several studies have examined organ-specific late effects of locoregional RT in BC, the association between lung-specific symptoms and RT remains debated [7–9]. To our knowledge, patient-reported RT toxicity and dose–response relationships between radiation lung doses and respiratory symptoms using PROMs have not been reported.

The Danish Breast Cancer Group (DBCG) was established in 1976 with the purpose of collecting data on patient, tumour, treatment and outcome characteristics, initiate research studies and develop national guidelines for BC therapy [10]. As part of the DBCG RT Nation Study, CT-based RT plans from 7,448 high-risk BC patients treated during 2008–2016 according to the DBCG RT guidelines were collected [11]. From these plans, individual data on doses to organs at risk, including the lungs, were available, enabling a more detailed understanding of the risk of respiratory late effects from loco-regional RT.

The Danish Patient Association of Late Effects [12] has received numerous inquiries from BC survivors attributing respiratory insufficiency to prior RT. In many cases, no diagnostic evaluation was performed, as symptoms were presumed irreversible and RT-related. Radiation-induced lung injury results from irradiation of healthy lung tissue and typically presents as early-onset pneumonitis or late-onset fibrosis. Radiation fibrosis is a chronic, irreversible condition managed only symptomatically. Risk factors include patient-related variables (age, smoking, comorbidity) and treatment-specific factors (irradiated lung volume, total dose, dose per fraction [fx]) [13–15]. This has led some general practitioners to attribute dyspnoea and cough to prior RT, often assuming it to be permanent.

Previous large-scale studies [16, 17] have demonstrated dose–response relationships when investigating risk of heart disease in relation to estimated mean heart dose, and risk of secondary cancer related to estimated mean lung doses (MLDs) after adjuvant BC RT. This study aimed to assess whether locoregional BC RT is associated with respiratory symptoms. We hypothesised that, if RT was causative for lung symptoms, higher radiation doses would correlate with increased patient-reported symptoms, indicating a dose–response relationship. The primary objective was to evaluate the prevalence of pulmonary symptoms using PROMs in high-risk BC patients treated with locoregional RT.

## Patients/material and methods

### Participants

Eligible patients were identified in the DBCG database. All patients had been treated with loco-regional RT from 2008 to 2016 in the Central Region of Denmark and were alive without BC recurrence or other malignancies. They were all included in the DBCG RT Nation study, thus the RT plan was available for analysis. All patients had high-risk BC with macro-metastatic nodal disease.

The study was approved by The Central Danish Region (no. 1-45-70-86-21).

### Patient reported outcome measures

Eligible BC survivors were contacted electronically using the Danish eBoks system allowing secure digital communication. They received an online questionnaire generated in REDCap [18], which included selected items from validated questionnaires. The PROs *cough* and *dyspnoea* were selected as they were the symptoms reported by patients contacting the Danish Patient Association of Late Effects. Furthermore, it was established in collaboration with a professor of pulmonary diseases (EB) that they are representative symptoms of pulmonary damage.

The item regarding the possible presence of dyspnoea was obtained from EORTC QLQ-C30 [19, 20] and questions regarding the severity (the sensory-perceptual dimension) and the degree to which dyspnoea and coughing disturbed activities of daily living (interference) were obtained from the PRO-CTCAE questionnaire [21, 22]. The presence of dyspnoea was rated on a 4-point Likert scale (‘not at all’, ‘a little’, ‘some’, ‘a lot’), and coughing severity on a 5-point Likert scale (‘none’, ‘mild’, ‘moderate’, ‘severe’, ‘very severe’). In addition, to assess possible confounders of the association between RT dose and respiratory symptoms, the questionnaire included questions on height, weight, smoking status, chronic obstructive pulmonary disease (COPD), asthma and heart disease taken from the questionnaires used in the DBCG Skagen trial 1 (NCT02384733). Patient reported COPD and asthma were reported collectively as ‘obstructive lung disease’. Disease and treatment characteristics were identified in the DBCG database [23]. PROMs were scored according to the official scoring manuals provided for the respective instruments [24, 25] and transformed to a 0–100 scale following the scoring guidelines, with higher scores indicating worse symptoms. All scoring and transformations were performed using standardized procedures for consistency and comparability.

The responses regarding the severity of dyspnoea were compared to normative data for patients with BC. Responders were separated into tertiles based on MLD (‘low’/’intermediate’/’high’) and dichotomised based on dyspnoea (*low* group: ‘no/little’ vs. *high* group: ‘quite a bit/very much’) and coughing (*low* group: ‘none/mild’ vs. *high* group: ‘moderate/severe/very severe’). The PRO variables were originally collected as categorical data and were analysed as such. Some PRO variables were converted to numerical data in order to report data, examine directions and compare with normative values. Lung dose was categorised in tertiles in order to investigate whether higher doses were associated with more severe symptoms. Tertiles were chosen as the smallest number of categories that would reveal a potential direction in the PRO results.

Every reference to dyspnoea, cough or comorbidities will henceforth be patient-reported.

For the respondents in the *high* dyspnoea group, all patient files were screened with regard to possible pre-disposing conditions (cardiac or pulmonary comorbidities) at the time of the questionnaire response, as well as for imaging results revealing signs of COPD or interstitial lung disease.

### RT planning

Details on treatment planning in this cohort are provided elsewhere [11, 26–29]. All treatment plans were generated with CT imaging using 3D conformal technique. The majority of treatment plans had target volume delineation carried out according to the DBCG guidelines [30], and from 2015 according to ESTRO guidelines [31]. The nodal targets were in all patients: CTVn_L2-4, CTVn_interpectoral and for very high risk patients also CTVn_L1. Patients treated from 2008 through 2014 were part of the DBCG IMN study and received internal mammary node irradiation (IMNI) if the tumour was right-sided, but not if left-sided [4, 5, 32]. During 2014, the DBCG guideline was modified to include IMNI irrespective of laterality based on results from the DBCG IMN study and other trials [33, 34]. The lungs were automatically delineated. DBCG consensus constraints for the ipsilateral lung were the volume receiving 20 Gray (Gy) (V20) in 50Gy RT and V17 in 40Gy RT of maximum 35%, and for the heart, V40 (50Gy) and V35 (40Gy) of maximum 5%. The heart and pericardium were delineated according to DBCG guidelines, and constraints were V10heart max 20Gy, and V5heart max 40Gy. No constraints for the mean heart dose were used. Deep inspiration breath hold (DIBH) was introduced as a standard for left-sided treatment in 2012, and for right sided locoregional treatment in 2014 [11]. The vast majority were treated with 50Gy in 25 fx, few patients received 40Gy/15fx. Tumour bed boost was indicated after breast conserving surgery (BCS), comprising 10Gy/5fx for patients aged 41–49 years, and 16Gy/8fx for patients <41 years, or if tumour resection margin was < 2 mm. Few patients had simultaneous integrated boost (63Gy/50Gy/28fx, 57Gy/50Gy/25fx, 52.2Gy/42.3Gy/18fx, 45.75Gy/40Gy/15fx) [35].

Individual RT characteristics were collected using the methods described in the DBCG RT Nation Study [28], and the ipsilateral MLD, V20lung and V5lung as well as mean heart dose were registered.

### Surgery and adjuvant treatment

Surgery comprised BCS or mastectomy, depending on tumour size, tumour location, co-morbidity and the patient’s genetic status. Axillary node surgery was performed per DBCG guideline as axillary lymph node dissection (ALND) or sentinel node (SN) operation, followed by ALND if there was minimum one macrometastasis. From 2015, however, some patients with macrometastases in 1–2 SN were randomised in the Senomac trial [36] between ALND versus no ALND.

All patients were considered high-risk due to nodal disease, and systemic therapy was prescribed according to guidelines. Chemotherapy was administered as neo-adjuvant or adjuvant treatment, all regimens contained anthracyclines and taxanes, and patients with HER2 positive BC received HER2 targeted treatment. Patients with hormone receptor positive BC had 5 years letrozole (postmenopausal) or 10 years tamoxifen (pre-menopausal) [11].

### Statistics

Descriptive statistics were used to summarise patient characteristics, RT dose–volume histogram (DVH) parameters and responses from the questionnaires. For each group (low/intermediate/high dyspnoea, low/high dyspnoea and cough), a separate table was generated to compare patient characteristics, DVH value, and PROs across categories. Additional analyses, that is logistic regression were not carried out due to the relatively small number of events.

We used the TableOne Python package [37] to generate summary tables, including means and standard deviations (SDs) for normally distributed continuous variables, medians and interquartile ranges for skewed distributions and counts with percentages for categorical variables. The package’s built-in statistical tests were applied to assess differences between groups: Chi^2^ or Fisher’s exact tests for categorical variables, and two-sample t-tests, Kruskal-Wallis or one-way ANOVAs for continuous variables, depending on data distribution.

All analyses were conducted in Python (3.12), using pandas 2.2.1 and TableOne 0.8.0 [37].

## Results

**Figure 1:**
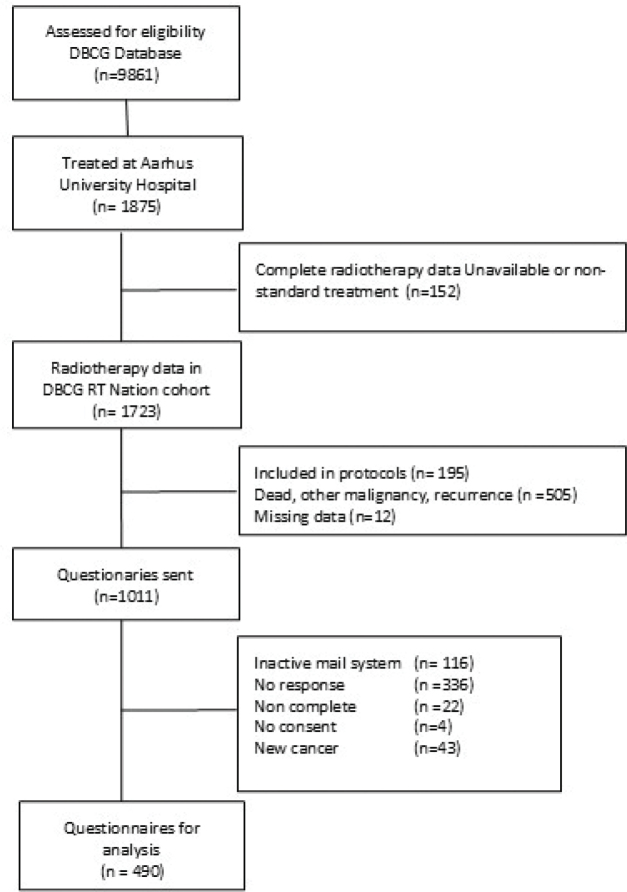
Consort diagram illustrating the inclusion of patients.

A total of 1,011 questionnaires were distributed in March 2023, and the responses were received from March to June 2023. A total of 537 (53%) completed the questionnaire, with 490 (49%) having no new cancer or recurrence, thus being available for analysis (Figure 1). The median age of responders was 55.7 years (interquartile range [IQR] 48.1;61.7) at the time of RT and 65.8 years (IQR 58.8;73.4) when completing the questionnaire. Median time from RT was 11.1 years (IQR 8.9;13.2). Regarding smoking status, 210 (45%) were never smokers and 259 (55%) were former (209, 45%) or current (50, 11%) smokers. A total of 47 (10.2%) responders reported obstructive lung disease including asthma or COPD, and 54 (11.9%) responders reported heart disease. Mean body mass index (BMI) was 26.6 (SD 4.9). Table 1 provides data on patient, tumour and treatment characteristics.

**Table 1:** Patient characteristics of all responders.

Patient characteristics		*N* (%)
Overall		490 (100)
Age at radiotherapy, median (IQR)		55 (48.1;61.7)
Age at questionnaire, median (IQR)		65.8 (58.8;73.4)
Tumor size, *n* (%)	0–20 mm	258 (55)
	21–50 mm	196 (41)
	>50 mm	18 (4)
No of positive lymph nodes, *n* (%)	1–3	320 (69)
	4–9	126 (27)
	≥10	19 (4)
Histologic type, *n* (%)	IDC	409 (84)
	ILC	46 (9)
	Other	35 (7)
HER2 status, *n* (%)	Positive	88 (18)
	Negative	402 (82)
Type of surgery, *n* (%)	Lumpectomy	273 (56)
	Mastectomy	215 (44)
Chemotherapy, *n* (%)	Not given	112 (23)
	Yes	378 (77)
Endocrine therapy, *n* (%)	Not given	73 (15)
	Yes	417 (85)
HER2 targeted therapy, *n* (%)	Not given	418 (85)
	Yes	72 (15)
Smoking, *n* (%)	Current/former smoker	259 (55.2)
	Never smoker	210 (44.8)
	Missing	21
Body mass index, mean (SD)		26.6 (4.9)
	Missing	28
Obstructive lung disease, *n* (%)	No	414 (89.8)
	Yes	47 (10.2)
	Missing	22
Heart disease, *n* (%)	Yes	398 (88.1)
	No	54.9 (11.9)
	Missing	38

RT: Radiotherapy; IDC: Invasive ductal carcinoma; ILC: Invasive lobular carcinoma.

**Figure 2:**
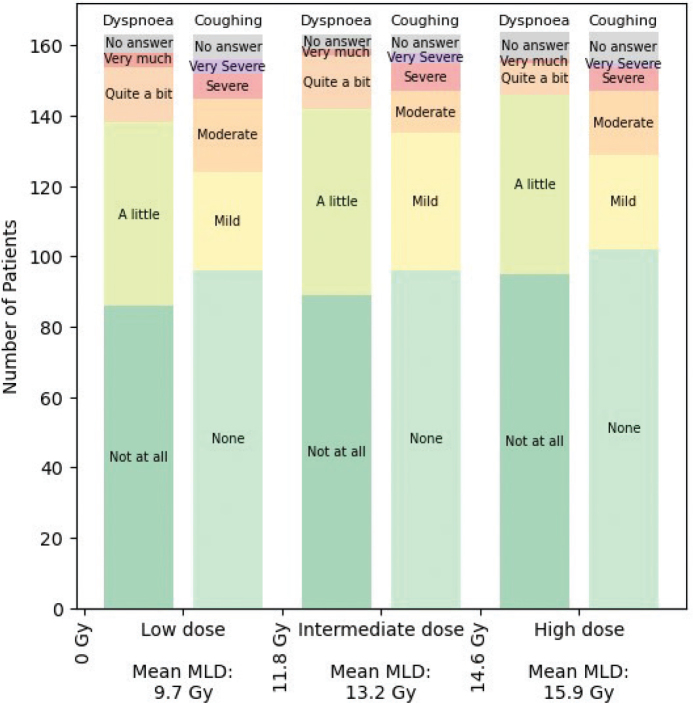
The distribution of patient-reported dyspnoea and cough in the low, intermediate and high lung dose groups. MLD: Mean lung dose; Gy: Gray.

The overall MLD was 12.9Gy (SD 2.8). The MLD limits between the low-, intermediate- and high dose groups were 11.8Gy and 14.6Gy, respectively. Figure 2 shows the distribution of dyspnoea and cough scores in the three MLD groups and mean dyspnoea score per group. Table 2 displays dyspnoea and cough scores with treatment characteristics in the low-, intermediate- and high MLD groups. While MLD decreased slightly with more dyspnoea, no significant associations between dyspnoea and low/intermediate/high MLD (*p* = 0.18) were found. No associations between dyspnoea score and smoking status, BMI, heart disease or lung disease were observed.

**Table 2:** Dyspnoea and cough scores in patients receiving low, intermediate and high lung dose to the ipsilateral lung.

		Overall	Low dose	Intermediate dose	High dose	*P*
*n*		490	163	163	164	
Dyspnoea score, mean (SD)		17.9 (23.6)	20.1 (25.6)	18.5 (23.7)	15.2 (21.0)	0.18
	Missing	17				
Cough score, mean (SD)		15.7 (24.2)	17.1 (25.8)	15.7 (24.0)	14.2 (22.8)	0.56
	Missing	21				
Mean lung dose [Gy], mean (SD)		12.9 (2.8)	9.7 (1.5)	13.2 (0.8)	15.9 (0.8)	<0.001
Mean heart dose [Gy], mean (SD)	Left	2.2 (1.1)	1.9 (0.9)	2.6 (1.3)	2.4 (1.1)	0.05
	Right	0.9 (0.8)	0.74 (0.3)	0.80 (0.4)	1.0 (1.0)	
Laterality, *n* (%)	Left	236 (48.2)	132 (81.0)	73 (44.8)	31 (18.9)	<0.001
	Right	254 (51.8)	31 (19.0)	90 (55.2)	133 (81.1)	
Gating, *n* (%)	No	337 (68.8)	85 (52.1)	124 (76.1)	128 (78.0)	<0.001
	Yes	153 (31.2)	78 (47.9)	39 (23.9)	36 (22.0)	

SD: standard deviation.

A total of 203 patients (41%) reported any degree of dyspnoea. The overall mean dyspnoea score was 17.9 (SD 23.6). Dyspnoea was dichotomised in none/little (low dyspnoea group, *n* = 443) versus quite a bit/very much (high dyspnoea group, *n* = 47) (Table 3). The mean dyspnoea score in the low dyspnoea group was 12.9 (SD 15.9), and the mean score in the high dyspnoea group was 71.1 (SD 12.2). When evaluating the ipsilateral lung dose in patients in the two groups, V20lung was 25.2% (low dyspnoea) and 23.2% (high dyspnoea) (*p* = 0.06) and V5lung was 45.0 and 42.3%, respectively (*p* = 0.04). Correspondingly, MLD was higher in the low dyspnoea group (13.0Gy vs. 12.0Gy, *p* = 0.04). Dyspnoea score was significantly higher in patients who reported obstructive lung disease (asthma or COPD) versus no lung disease (*p* < 0.001), whilst no differences in smoking status, BMI or heart disease were present.

**Table 3:** Patient and treatment factors according to low/high dyspnoea scores.

		Overall	Low dyspnoea score	High dyspnoea score	*P*
*n*		490	443	47	
Dyspnoea score, mean (SD)		17.9 (23.6)	12.1 (15.9)	71.1 (12.2)	<0.001
	Missing	17			
Cough score, mean (SD)		15.7 (24.2)	11.8 (20.1)	50.0 (30.4)	<0.001
	Missing	4			
Mean lung dose [Gy], mean (SD)		12.9 (2.8)	13.0 (2.7)	12.0 (3.0)	0.04
Ipsilateral lung V20 Gy [%], mean (SD)		25.2 (6.4)	25.4 (6.3)	23.2 (6.9)	0.04
Ipsilateral lung V5 Gy [%], mean (SD)		44.7 (8.2)	45.0 (8.2)	42.3 (8.2)	0.04
Mean heart dose [Gy], mean (SD)		1.5 (1.1)	1.6 (1.2)	1.4 (0.8)	0.19
Laterality, *n* (%)	Left	229 (48.4)	205 (48.1)	24 (51.1)	0.82
	Right	244 (51.6)	221 (51.9)	23 (48.9)	
Gating, *n* (%)	No gating	321 (67.9)	289 (67.8)	32 (68.1)	1.00
	Gating	152 (32.1)	137 (32.2)	15 (31.9)	
Smoking, *n* (%)	Ever smoked	259 (55.2)	230 (54.4)	29 (63.0)	0.33
	Never smoked	210 (44.8)	193 (45.6)	17 (37.0)	
	Missing	4			
Obstructive lung disease, *n* (%)	No	429 (93.3)	390 (94.0)	39 (86.7)	0.11
	Yes	31 (6.7)	25 (6.0)	6 (13.3)	
	Missing	30			
Heart disease, *n* (%)	No	398 (88.1)	364 (89.0)	34 (79.1)	0.10
	Yes	54 (11.9)	45 (11.0)	9 (20.9)	
	Missing	38			
Body mass index, mean (SD)		26.6 (4.9)	26.5 (4.7)	27.8 (6.5)	0.19
	Missing	28			

SD: standard deviation; Gy: Gray; V20: Volume receiving 20% of the prescribed dose; V5: Volume receiving 5% of the prescribed dose.

A total of 175 survivors (37%) reported any degree of coughing. The overall mean cough score was 15.7 (SD 24.2). Coughing was dichotomised in a low cough group (not at all/a little bit) (*n* = 388) versus high cough group (moderate/quite a bit/very much) (*n* = 81) (Table 4), with mean cough scores in the low cough group of 6.1 (SD 10.7) and 61.7 (SD 16.8) in the high cough group. MLD in the two groups did not differ (13.0 Gy vs. 12.5 Gy, *p* = 0.23), nor did V5lung (45.0% vs. 43.8%, *p* = 0.25), or V20lung (25.3% vs. 24.2%, *p* = 0.19).

**Table 4:** Patient and treatment factors according to low/high cough scores.

		Overall	Low cough score	High cough score	*P*
*n*		469	388	81	
Cough score, mean (SD)		15.7 (24.2)	6.1 (10.7)	61.7 (16.8)	<0.001
Dyspnoea score, mean (SD)		18.1 (23.6)	13.7 (19.9)	39.2 (28.3)	<0.001
Mean lung dose [Gy], mean (SD)		12.9 (2.8)	13.0 (2.7)	12.5 (3.1)	0.23
Lung V20 Gy [%], mean (SD)		25.1 (6.4)	25.3 (6.2)	24.2 (7.1)	0.19
Lung V5 Gy [%], mean (SD)		44.8 (8.2)	45.0 (8.2)	43.8 (8.4)	0.25
Mean heart dose [Gy], mean (SD)		1.5 (1.1)	1.6 (1.2)	1.2 (0.7)	<0.001
Laterality, *n* (%)	left	227 (48.4)	194 (50.0)	33 (40.7)	0.16
	right	242 (51.6)	194 (50.0)	48 (59.3)	
Gating, *n* (%)	False	320 (68.2)	267 (68.8)	53 (65.4)	0.64
	True	149 (31.8)	121 (31.2)	28 (34.6)	
Smoking, *n* (%)	Ever smoked	259 (55.7)	208 (54.2)	51 (63.0)	0.19
	Never smoked	206 (44.3)	176 (45.8)	30 (37.0)	
	Missing	4			
Obstructive lung disease, *n* (%)	No	411 (89.7)	351 (92.6)	60 (75.9)	<0.001
	Yes	47 (10.3)	28 (7.4)	19 (24.1)	
	Missing	11			
Heart disease, *n* (%)	No	394 (87.9)	335 (90.5)	59 (75.6)	<0.001
	Yes	54 (12.1)	35 (9.5)	19 (24.4)	
	Missing	21			
Body mass index, mean (SD)		26.6 (4.9)	26.3 (4.7)	28.2 (5.8)	0.006
	Missing	11			

Gy: Gray; V20: Volume receiving 20% of the prescribed dose; V5: Volume receiving 5% of the prescribed dose.

A secondary analysis of the 47 patients reporting moderate or severe dyspnoea showed that 25 (53%) had cardiac or pulmonary co-morbidity with dyspnoea as a cardinal symptom (asthma, COPD, heart failure, atrial fibrillation) or conditions potentially explaining the dyspnoea (intrathoracic strumae pressing on trachea, laryngeal swelling). Computer Tomography scan of 34 patients revealed six patients with ground glass opacities not related to previous RT. Eight patients (17%) were treated with inhalation medication prescribed for asthma/dyspnoea by the general practitioner.

## Discussion

The present study evaluated patient-reported respiratory symptoms more than a decade after treatment and explored if these symptoms were likely associated with adjuvant locoregional RT for high-risk BC. Overall, 30–40% of the patients reported dyspnoea and coughing, however, these symptoms did not show a correlation to high radiation dose to the lung. Previous studies of the influence from RT on pulmonary function have used different endpoints and reported inconsistent results.

The pathogenesis of radiation induced lung injury is complex, involving multiple cell types of the lungs and the immune system with the involvement of damage-associated-molecular-pathways, cytokines and chemokines released through complex pathways from dying or senescent cells [15]. Predictive factors for developing pulmonary toxicity after adjuvant BC RT include age, pulmonary comorbidity, smoking and importantly, dosimetric factors, including total dose, dose per fraction and irradiated lung volume [13–15]. Diagnosing pulmonary late toxicity after BC RT is challenging; BC treatment is comprehensive and often involves different systemic treatments in addition to RT. In addition, symptoms such as dyspnoea and cough are not specific for treatment induced pulmonary damage, and may be caused by specific pulmonary comorbidities, lifestyle factors or comorbidities in other organ systems (e.g. heart disease). However, one could expect that if RT led to worse pulmonary function, the association would show a dose response relationship, which has been demonstrated e.g. in radiation associated heart disease and second lung cancer [16, 17]. This study aimed to show a dose–response relationship, but did not show such a relation. This is noteworthy, since the study was conducted at the largest Danish centre irradiating BC patients, and all patients treated during an 8-year period were invited to participate. Still, relatively few pulmonary events were detected, thus limiting the scope of statistical analyses.

Other studies have investigated respiratory symptoms after BC RT. A Norwegian study investigated respiratory function in 250 survivors receiving adjuvant local or locoregional RT for BC [7], where they collected PROs and performed imaging and objective examination of pulmonal function. Overall, 35% reported any degree of dyspnoea, but no significant associations with imaging or objective pulmonary function were observed. Unlike our study, they included both local and locoregional RT but did not assess etiological factors or RT dose-volume parameters. The CANTO-RT study [8] comprised prospective data in a large cohort of early BC patients receiving adjuvant RT. Of 1,565 patients, 712 (46%) reported cough and/or dyspnoea within 5 years after RT. A total of 38 (2.4%) developed radiation induced lung injury, and unlike the current study, they found an unambiguous association with radiation dose to the ipsilateral lung (MLD, lungV5-V40). Normative EORTC QLQ-C30 data from over 15,000 individuals across 13 countries reported a mean dyspnoea score of 16.6 (SD 25.0) for ages 60–65. Our cohort showed a comparable mean of 17.9, indicating alignment with general population norms.

Lung dose constraints are defined in the DBCG guidelines and are generally well-adhered to in clinical practice. Different dosimetric parameters are highly correlated, and the parameter for reporting pulmonary dose varies. The DBCG uses V20lung, V5lung represents the low dose bath. We report both MLD, V5lung and V20lung, as it increases both the clinical applicability and the relevance of the study. De Rose et al. [9] recently reviewed dose limits in BC RT, highlighting the fact that ipsilateral lung dose increases with treatment complexity. They recommend a V17/V20 constraint of 25% in patients without a periclavicular target, while the American Society for Radiation Oncology (ASTRO) suggests a stricter limit of <15%. DBCG allows V17/V20 of 35% for locoregional treatment. In the present study, the V20lung was 25.2%, aligning well with international recommendations.

In this study, a significantly higher lung dose was observed among responders with right-sided tumours. This may be explained by the inclusion of IMN RT in right-sided tumours in the DBCG IMN2 study [5]. Additionally, respiratory gating was initially implemented for left-sided BC to reduce cardiac exposure and only later adopted for right-sided treatment. The combination of IMN RT and later introduction of gating likely contributed to the increased lung doses in right-sided patients. Meanwhile, DBCG has prioritised minimising radiation dose to the heart, and data from this cohort on cardiac exposure have been reported previously [5, 11], showing generally low mean heart doses, likely due to the widespread use of respiratory gating.

To our knowledge, no studies have evaluated PROMs and compared the results with the corresponding treatment plan. A limitation of our study is the lack of systematic examinations of objective pulmonary function and imaging. Patient-reported pulmonary co-morbidity might be subject to bias based on patients not consulting the general practitioner with novel respiratory symptoms, time that passes between the first symptoms to the diagnosis of pulmonary disease or the stigma, for example the diagnosis COPD due to use of tobacco, that may be neglected or misjudged as asthma. The prevalence of patient-reported versus general practitioner diagnosed asthma has been investigated in a review, showing that rates of self-reported asthma among children and adolescents are substantially higher than asthma diagnosed by a physician. In Denmark, the prevalence of COPD among the 65–79-year-olds in a large epidemiologic study was 23.9% [38]. Our data reported COPD and asthma in one category (obstructive lung disease), with a prevalence of 10.2%. PROMs may represent cases of undiagnosed/untreated respiratory symptoms. It is important to note that patients with severe dyspnoea or known pulmonary risk at the time of BC diagnosis, would undergo primary mastectomy and ALND to omit adjuvant RT and therefore be excluded from this cohort (< 5% of early BC cases). Despite this, our study design facilitates individualised insights, enhancing prediction of outcomes in future BC patients and a response rate of ~50% is high among long-term survivors and supports the external validity of our findings. A small number of patients reporting moderate/severe dyspnoea were reviewed, and for 25 participants, an obvious reason for the respiratory symptoms was present. Yet, they were included in the final analysis, as they add to the representation of the population encountered in daily clinical practice, with some individuals having pre-existing conditions that may predispose them to developing respiratory symptoms. In practice, such conditions do not preclude adjuvant RT, and excluding these patients would therefore reduce the clinical relevance of the results. Furthermore, pulmonary damage following RT is currently a diagnosis of exclusion, and among patients reporting dyspnoea, other undiagnosed conditions may also contribute to the symptoms. In addition, the reporting of pulmonary diagnoses in this study was based on the status at median 11 years after RT, since it was not possible to retrieve pulmonary diagnoses at the time of RT. A limitation of the study is the lack of data from non-responders, which we were not able to report due to restricted study approval regulations.

A shift in Danish BC follow-up is underway, with a move toward fewer hospital visits and increased use of PROMs to enhance patient involvement. In response, the DBCG Centre and Clinic for Late Effects (DCCL) has been established to systematically collect PROMs from diagnosis to death (manuscript submitted). This initiative will soon provide annual, prospective data for all Danish BC patients, enabling more detailed analyses of late effects, including respiratory symptoms.

RT improves overall survival in high-risk BC patients, and some degree of late effects is therefore acceptable. Understanding the patterns of late effects is essential to inform, detect and manage them appropriately. Equally important is distinguishing symptoms unrelated to cancer treatment to avoid patient misinformation and suboptimal care. In this study of a large, consecutive cohort of high-risk BC patients treated with modern systemic therapy, we found that respiratory symptoms were unlikely to be attributable to RT. These patients should undergo thorough diagnostic evaluation to ensure appropriate management.

## Data Availability

As data contain personal information, they cannot be accessed due to approval regulations.
